# Evidence for Positive Selection on a Number of MicroRNA Regulatory Interactions during Recent Human Evolution

**DOI:** 10.1371/journal.pgen.1002578

**Published:** 2012-03-22

**Authors:** Jingjing Li, Yu Liu, Xiaofeng Xin, Taehyung Simon Kim, Eduardo Aguiar Cabeza, Jie Ren, Rasmus Nielsen, Jeffrey L. Wrana, Zhaolei Zhang

**Affiliations:** 1 Banting and Best Department of Medical Research, The Donnelly Centre, University of Toronto, Toronto, Canada; 2 Department of Molecular Genetics, University of Toronto, Toronto, Canada; 3 The Center for Systems Biology, Samuel Lunenfeld Research Institute, Mount Sinai Hospital, Toronto, Canada; 4 Department of Physics and Centre for Computational Science and Engineering, National University of Singapore, Singapore, Singapore; 5 Departments of Integrative Biology and Statistics, University of California Berkeley, Berkeley, California, United States of America; University of Michigan, United States of America

## Abstract

MicroRNA (miRNA)–mediated gene regulation is of critical functional importance in animals and is thought to be largely constrained during evolution. However, little is known regarding evolutionary changes of the miRNA network and their role in human evolution. Here we show that a number of miRNA binding sites display high levels of population differentiation in humans and thus are likely targets of local adaptation. In a subset we demonstrate that allelic differences modulate miRNA regulation in mammalian cells, including an interaction between miR-155 and *TYRP1*, an important melanosomal enzyme associated with human pigmentary differences. We identify alternate alleles of *TYRP1* that induce or disrupt miR-155 regulation and demonstrate that these alleles are selected with different modes among human populations, causing a strong negative correlation between the frequency of miR-155 regulation of *TYRP1* in human populations and their latitude of residence. We propose that local adaptation of microRNA regulation acts as a rheostat to optimize *TYRP1* expression in response to differential UV radiation. Our findings illustrate the evolutionary plasticity of the microRNA regulatory network in recent human evolution.

## Author Summary

MicroRNAs (miRNAs) are endogenous small RNAs that bind to their target mRNAs to post-transcriptionally repress protein production. miRNA–mediated gene regulation is usually considered to be strongly conserved among and within species, and thus alteration of such regulations is usually considered as detrimental. However, it is likely that evolutionary divergence of miRNA regulation may actually be selectively advantageous and could even serve as a genetic reservoir for innovation and adaptation. Towards this goal, we identified a number of polymorphic miRNA binding sites that display extreme population differentiation and show evidence of positive selection. We experimentally validated 3 regulations, including a regulation by miR-155 on *TYRP1*, a melanosomal enzyme associated with human pigmentation. We found that the two alternate alleles on the 3′ UTR of *TYRP1*, either inducing or disrupting repression by miR-155, are under opposite selections among human populations. This results in a strong negative correlation between the degree of fixation of miR-155–mediated repression of *TYRP1* in a population and the population's latitude of residence. These observations collectively suggest miR-155 acts a rheostat to optimize *TYRP1* expression for local adaptation to differential UV radiation along the latitudes. Our findings demonstrate the plasticity of miRNA regulation in recent human evolution.

## Introduction

microRNAs (miRNAs) are endogenous small RNAs that bind to their target mRNAs to post-transcriptionally repress protein production. They recognize their target mRNAs primarily through sequence complementarity between the seed region of a miRNA (usually defined as the 2^nd^ to the 7^th^ or 8^th^ nucleotide along a miRNA 5′ end) and the binding sites on its target mRNAs [1]. A large number of human genes are known to be regulated by miRNAs, thus, miRNAs constitute a critical post-transcriptional regulatory network that plays vital roles in a broad range of biological processes [2]–[4]. Their functional importance is also consistent with the evolutionary conservation of miRNA-mediated regulations, as many miRNAs and their targets are conserved across species [5], [6] and sequence variants that disrupt miRNA regulation are typically rare in humans and are often associated with human diseases [7]–[9]. However, human phenotypic evolution can be caused by changes in gene regulation, perhaps even more so than by changes in the proteins themselves [10]–[13]. Modulation of miRNA regulations is a possible path for such adaptive changes, but hitherto no solid evidence has been presented in favor of miRNA interactions playing an important role in human evolution. To address this issue, we first examined the degree to which local human adaptation is affected by changes in miRNA regulatory interactions, and then experimentally verified some of the identified interactions showing extreme population differentiation, including the regulation by miR-155 on *TYRP1* to modulate human pigmentation phenotype.

## Results

### Differentiation of miRNA–mediated regulation among human populations

We first aim to identify miRNA regulatory interactions that have been significantly differentiated among human populations, and then determine whether these differentiation events were driven by positive selection during human evolution. We mapped the ∼3 million HapMap Phase II single-nucleotide polymorphisms (SNPs) [14] onto predicted binding sites of known human miRNAs using TargetScanS [15], and identified 2,217 bi-allelic SNPs that have one allele disrupting an intact binding site. We considered only those binding sites that have high-confidence scores (see Materials and Methods, and Figure S1A). Population differentiation of each SNP was then quantified by *F_ST_*, a commonly used statistical measure of genetic differentiation [16]. A SNP locus that has exlucively alternate alleles among populations receives an extreme *F_ST_* value. We used *F_ST_* computed by Barreiro *et al.* [17] for the 4 HapMap populations: Yoruba from Ibadan, Nigeria (YRI), Japanese from Tokyo (JPT), Han Chinese from Beijing (CHB) and Utah residents with northern and western European ancestry (CEU). Our subsequent analysis revealed that many SNPs showing extreme population differentiation interfere with their predicted miRNA regulatory interactions (Table S1). Given the short evolutionary history of human population differentiation, this finding is in contrast with the common thought that miRNA-mediated regulation is strongly conserved [5], [6].

### Positive selection on miRNA regulatory interactions

**Figure 1:**
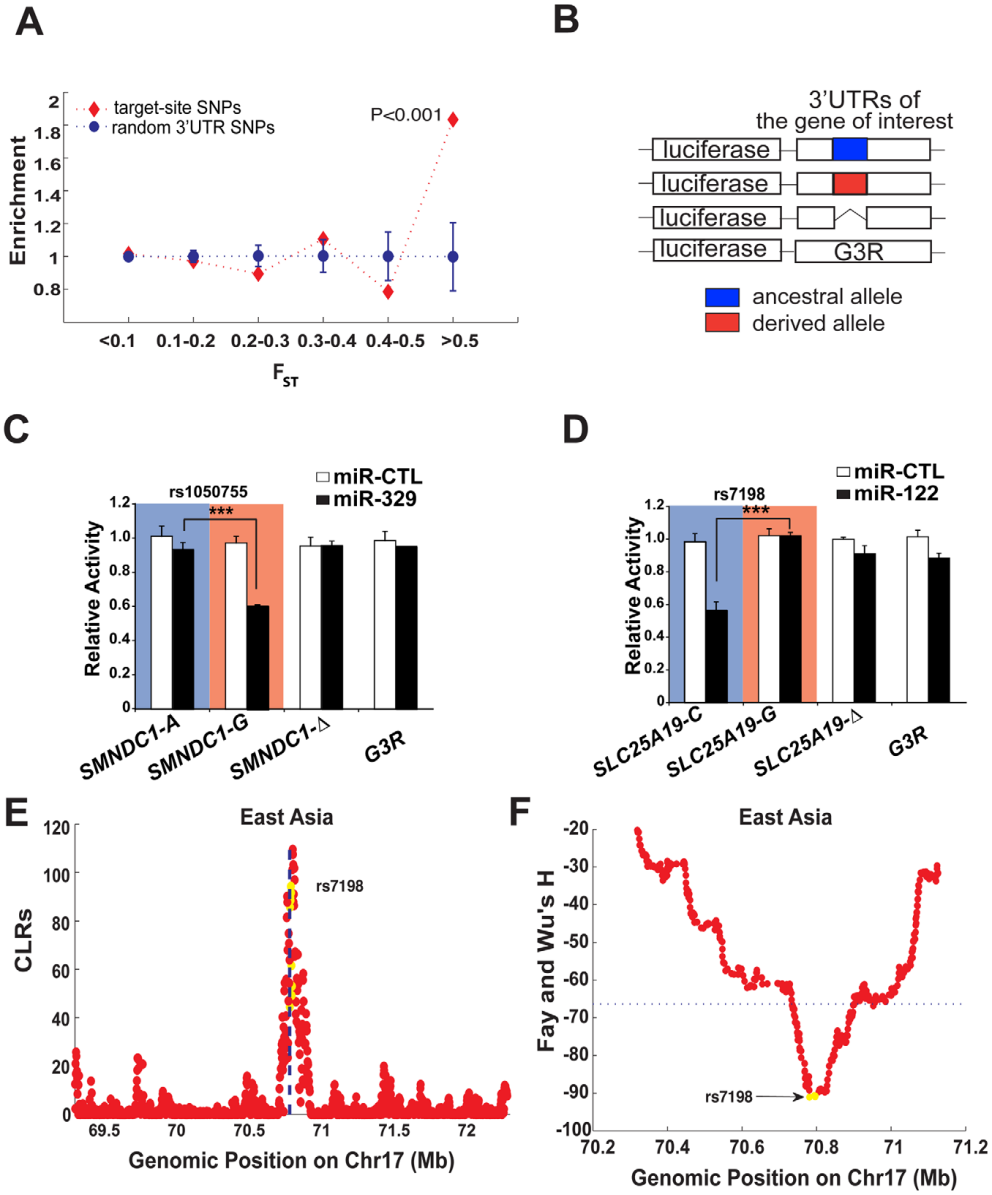
Identification of miRNA binding sites that show extreme population differentiation. (A) The predicted miRNA binding sites are significantly enriched among the High-*F_ST_* loci on 3′ UTRs. The enrichment score was the fraction of the target-site SNPs in each bin divided by the fraction of all the 3′ UTR SNPs in the same bin. Likewise the enrichment score for the randomly sampled SNPs in each bin was computed in the same way based on 1, 000 bootstrap resampling from all the 3′ UTR background SNPs. Error bar represents one standard deviation. (B–D) In vitro validation of the predicted miRNA binding sites showing extreme population differentiation. The 3′ UTR of genes selected for validation were cloned into the pMIR-REPORTER vector. Variants of each gene harbouring the ancestral (blue), derived (red) alleles or a deletion of putative miRNA site were generated and analyzed along with the G3R control vector (B). For analysis of miRNA targeting, the reporters containing the indicated ancestral (blue shading) or derived (red shading) alleles of *SMNDC1* (C) or *SLC25A19* (D), as well as the deletants or the G3R control were transfected into HEK293T cells in the presence of either control miRNA (miR-CTL; white bars) or the relevant miRNA (black bars), as shown. 40-hour post-transfection, luciferase expression in cell lysates was measured by chemiluminescence and is plotted as activity relative to miR-CTL transfected cultures. Bars are the mean ± standard deviation for triplicate experiments and *** indicates P<0.001. (E–F) Statistical tests for positive selection on the miR-122 binding site on *SLC25A19* in East Asians (CHB+JPT), where the derived allele of rs7198 that compromises miR-122 regulation has reached high frequency in East Asians. The yellow dots represent genic position on *SLC25A19*, and the flanking non-genic positions are in red. The CLR test (E, where the dotted line indicates the locus of rs7198) and Fay-Wu's H statistic (F, where the dotted line indicates the 5% extreme value among the genome-wide SNPs) were used.

Extreme population differentiation can be attributed to several possible factors, such as outliers from neutral drift, population structure, or positive selection for local adapation. Thus we sought to determine the potential sources accounting for the observed differentiation events. Recent population genetic analyses of human variation have shown that much of the recent local adaptation in humans may be caused by subtle changes in allele frequencies in many genes rather than strong changes in a few genes [18], [19]. The effect of local (population-specific) selection can then be detected by comparing genome-wide pattern of *F_ST_*. For example, Coop *et al.* showed that SNPs with high *F_ST_* values in humans are enriched for genic SNPs in comparison to non-genic SNPs, and interpreted this as evidence of local selection targeting sites in genic regions [18]. Inspired by this work, we here ask whether a similar enrichment exists for miRNA target sites compared to the background distribution. Since the miRNA binding sites in this analyses were on the 3′ UTRs of human genes, we then collected a total of 23,030 3′ UTR SNPs to serve as a background control. We divided *F_ST_* values into different bins (Figure 1A), and computed the enrichment scores for SNPs in each bin. The enrichment score is the fraction of miRNA target-site SNPs in each bin, divided by the fraction of all 3′ UTR SNPs in the same bin. To determine the distribution of the enrichment score under the null hypothesis of no enrichment, we generated 1,000 data sets with 2,217 randomly sampled 3′UTR SNPs. Figure 1A clearly shows that loci with extreme *F_ST_* values (*F_ST_*≥0.5) were significantly enriched for miRNA binding sites (P<2.6×10^−4^; hypergeometric tail probability). Positive selection by definition acts on functional loci so these findings clearly indicate that miRNA binding sites with extreme population differentiation (*F_ST_*≥0.5) are likely targets of positive selection.

### Selection of candidate miRNA target site SNPs

We next focused on the polymorphic binding sites that have the strongest evidence of population differentiation (*F_ST_*≥0.5); however we also attempted to control, at least partially, for hitchhiking effects by excluding target-site SNPs linked to any annotated functional variant that lies 500 Kb upstream or downstream of the SNPs (following the protocol of HapMap, see Materials and Methods). Using these filters, we identified 30 SNPs located in the putative miRNA binding sites of 26 genes, which showed very strong evidence of population differentiation (Figure S1B, also see Tables S1 and S2). In addition to using *F_ST_* for cross-population comparison, we also examined these 30 loci for evidence of selection within individual populations, using several other aspects of the data including haplotype structure (the integrated haplotype scores, iHS [20]) and excessive high-frequency derived alleles (Fay-Wu's H test [21]). These revealed that approximately half of the identified SNPs showed evidence of selection using either test statistic (Table S1). To further elucidate whether selection has been acting directly on the target site, rather than on linked sites, we assessed composite likelihood ratios (CLR) to identify the location of a selective sweep [22]. Figure S2 shows several examples where selection signals can be clearly localized around the polymorphic binding sites using this method. While we cannot exclude that the particular sites identified using these analyses show high *F_ST_* values due to linkage with other SNPs that have not been annotated, or in a few cases are false positives with high degree of differentiation due solely to genetic drift, the procedures we have used here are designed to maximize the probability that the candidate sites identified here are targets of selection. Based on these studies we chose 7 candidate loci for further functional analyses (Table S3).

### In vitro validation of the differentiated miRNA regulations

**Figure 2:**
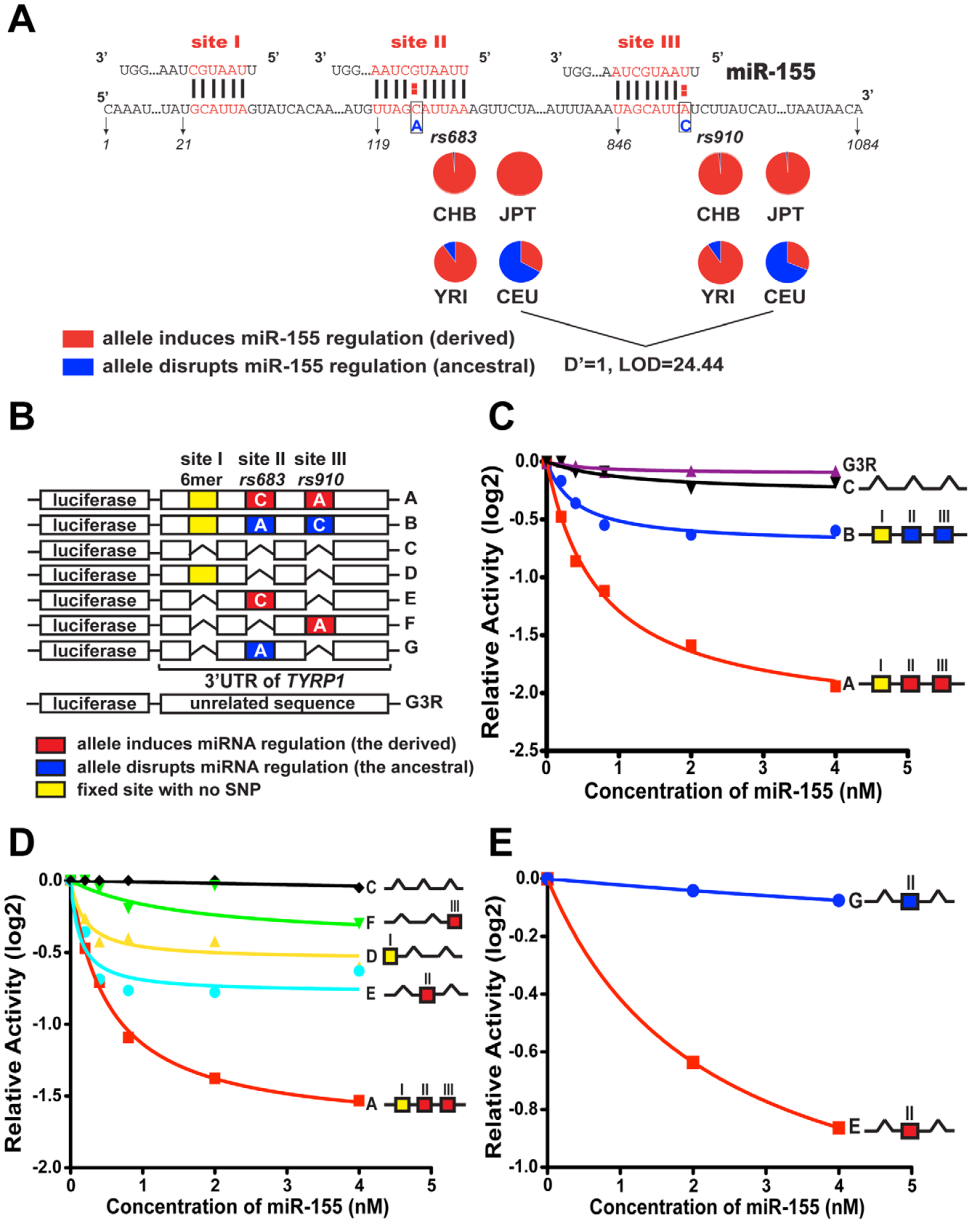
Modulation of miR-155 regulation of *TYRP1* by rs683 and rs910. (A) 3′ UTR sequences of *TYRP1*, which harbors 3 predicted miR-155 binding sites (site I, II and III). Site I had been fixed in all populations, mediating a 6-mer match to miR-155. The positions of SNPs rs683 and rs910 in sites II and III are shown with ancestral allele in blue and derived allele in red. The pie charts show the distribution of ancestral and derived alleles in CEU, CHB, JPT and YRI. Note that the derived alleles are almost fixed in CHB, JPT and YRI, but are highly polymorphic in CEU. (B–D) Effects of rs683 and rs910 on miR-155 regulation using luciferase reporters. Luciferase reporter constructs (shown in B) were individually transfected into HEK293T cells together with either miR-CTL or increasing concentrations of miR-155 as indicated. The luciferase expression in cell lysates was then measured by chemiluminescence 40 hours after transfection, and is plotted as log2-transformed activity relative to miR CTL prior to curve fitting. (C) Comparison of miR-155-mediated suppression of the ancestral (blue), derived (red) and deleted (black) variants of the *TYRP1* 3′UTR with the unrelated G3R control (purple). (D) *TYRP1* variants containing no sites, individual derived sites, or all three sites, as indicated, were assessed for miR-155-dependent targeting. (E) Comparison of miR-155 suppression of the derived (red) allele of site II analyzed in isolation (as indicated) with the ancestral variant (blue). Note that derived site II alone, but not the ancestral variant, is substantially suppressed by miR-155.

To validate whether the 7 predicted candidate loci indeed display differentiated miRNA regulations, we made three variants of the 3′ UTR for each locus fused downstream of the firefly luciferase coding sequence using the pMIR-REPORTER vector (Figure 1B). The first two variants carried either the ancestral or derived allele of a SNP within the putative miRNA target, while the third deleted the entire site. In addition, we employed the unrelated G3R 3′ UTR sequence, which is derived from the chicken versican G3 domain, as a control. Each reporter was then transfected into HEK293T cells either with control miRNA (miR-CTL) or a miRNA mimic that corresponds to the predicted miRNA regulator. Among the 7 putative interactions examined, we found that the predicted sites on *SMNDC1* (survival motor neuron domain containing 1), *SLC25A19* (solute carrier family 25, member 19), and *TYRP1* (tyrosinase-related protein 1) showed significant miRNA-dependent inhibition of luciferase expression in an allele-specific manner (Figure 1C and 1D and Figure 2), while the remaining 4 genes showed no evidence of regulation (Figure S3). For example, the ancestral ‘A’ allele of SNP rs1050755 in *SMNDC1* interfered with miR-329 regulation (Figure 1C). This allele is the most common allele in East Asians (CHB and JPT) and CEU, but is rare in YRI, which almost exclusively possess the derived miR329-targeted ‘G’ allele (derived allele frequency is 0.98). In the same vein, the ancestral allele ‘C’ of SNP rs7198 mediated allele-specific regulation of *SLC25A19* by miR-122, while its derived ‘G’ allele prevented regulation (Figure 1D). Notably, the derived allele reaches a frequency around 90% in East Asian populations (CHB and JPT) but is rare in YRI (with derived allele frequency 0.02). The third validated interaction, between miR-155 and two linked SNPs mapped to the 3′UTR of *TYRP1* was subject to extensive analysis (Figure 2 and discussed below). Of note, given the difficulty in predicting miRNA targets and a lack of consensus among miRNA target prediction algorithms [23], it is encouraging that we could validate 3 of the 7 *in silico* predicted interactions. Regardless, these results verify that the highly differentiated SNPs in several cases have a direct impact on miRNA-mediated regulation.

Worthy of note, for two of the three validated sites (on *SLC25A19* and *TYRP1*), the signal of positive selection can be localized directly to the miRNA binding sites using the CLR method (Figure 1E). The alteration, rs7198 on *SLC25A19* disrupted miR-122 regulation and is affected by the selective sweep, almost fixing the derived allele in East Asians with a frequency of ∼90%. This is further supported by analyses using other methods including Fay and Wu's H (Figure 1F), where a sharp reduction in H suggests an excess of derived alleles fixed by the sweep around this locus. These signals, however, were absent in YRI and CEU (Figure S4), particularly in YRI where the derived allele is very rare. The second verified miRNA regulation with extreme population differentiation and strong evidence of selection acting directly on the miRNA binding sites, *TYRP1*, is more interesting since it is associated with an obvious population differentiated phenotype – pigmentation. *TYRP1* is an enzyme specifically expressed in melanocytes, which promotes melanin production and regulates pigmentation in skin, eyes and hair [24]–[26]. Mutations in *TYRP1* can cause oculocutaneous albinism type 3 (OCA3) [27], [28]. Furthermore, *TYRP1* is important for adjusting skin reflectance to protect against excessive UV exposure [29] and recent genome-wide association studies have consistently found this gene to be associated with differentiated pigmentation among populations [30]–[32]. Lastly, several studies have suggested that this gene has been under strong positive selection relating to adaptation to local environments [33]–[35]; however no causal variants have been identified. In our study, we discovered that *TYRP1* harbors 2 common SNP variants, rs683 and rs910 that reside in two putative miR-155 binding sites (Figure 2A). These two SNPs are nearly fixed for the derived allele in the African and Asian populations (YRI, CHB and JPT), but remain polymorphic in the European population (CEU). Notably rs683 is reported to be associated with difference in iris color among Europeans [30]. To investigate this link we next sought to systematically validate the role of these SNPs in population-specific miRNA regulation of *TYRP1*.

### miR-155 selectively regulates TYRP1 in human populations

miR-155 is very narrowly expressed among human tissues [36], [37]. Interestingly it is reported to be expressed in melanocytes [38], suggesting it may physically interact with *TYRP1*. Notably miR-155 is also an oncomir [38], [39], and is involved in cell signaling and dendritic development [40]–[43]. The 3′ UTR of *TYRP1* contains three putative miR-155 binding sites, among which two are polymorphic in the HapMap data analyzed here. As seen in Figure 2A, site I is a non-canonical site mediating a 6-mer match to the miRNA seed and is located immediately downstream of the stop codon, while sites II and III mediate canonical pairing with the intact seed region of miR-155. The two SNPs, rs683 and rs910, reside within site II and site III respectively, with the derived alleles forming intact miRNA sites in CHB, JPT and YRI. In contrast, two thirds of CEU individuals carry the ancestral alleles that disrupt miRNA-target interaction. Due to their physical proximity, the two SNPs are tightly linked with D' = 1 and LOD = 24.44, indicating their co-presence (or co-absence) in CEU individuals (Figure 2A).

To test the function of these putative miR-155 targets, 7 variants of the *TYRP1* 3′UTR harboring combinations of ancestral, derived and deleted miR-155 target elements were constructed in pMIR-REPORTER (Figure 2B). Transfection into HEK293T cells either alone or in the presence of increasing amounts of synthesized miR-155 revealed that the 3′ UTR harboring the derived alleles (construct A, Figure 2C) was substantially suppressed by miR-155. Furthermore, a mutant in which all three putative miR-155 targets were deleted (construct C, Figure 2C) showed minimal repression that was comparable to the G3R control (G3R, Figure 2C). In contrast, when we analyzed the ancestral alleles (construct B, Figure 2C), suppression was compromised, indicating that the sequence variations in sites II and III interfered with miR-155-dependent regulation of *TYRP1* (Figure 2C). The derived *TYRP1* 3′ UTR (construct A) is thus a target of miR-155. Analysis of the ancestral alleles revealed substantial suppression when compared to a mutant in which all three miR-155 targets were deleted (construct C, Figure 2C). This suggested that the fixed site I might be functional. Therefore, to determine the relative contributions of the three sites to miR-155 regulation of *TYRP1*, we next tested various combinations of site deletants (Figure 2B). This revealed that site II mediated the strongest suppression (Figure 2D, curve E), while site I was weaker (Figure 2D, curve D) and site III was the weakest (Figure 2D, curve F). As site III is linked with site II (Figure 2A), we subsequently analyzed alternate site II alleles in isolation, since the strongest suppression mediated by this site might serve to direct natural selection on this locus. We observed that interruption of the site by the ancestral allele (construct G) strongly blocked miR-155 suppression (Figure 2E).

**Figure 3:**
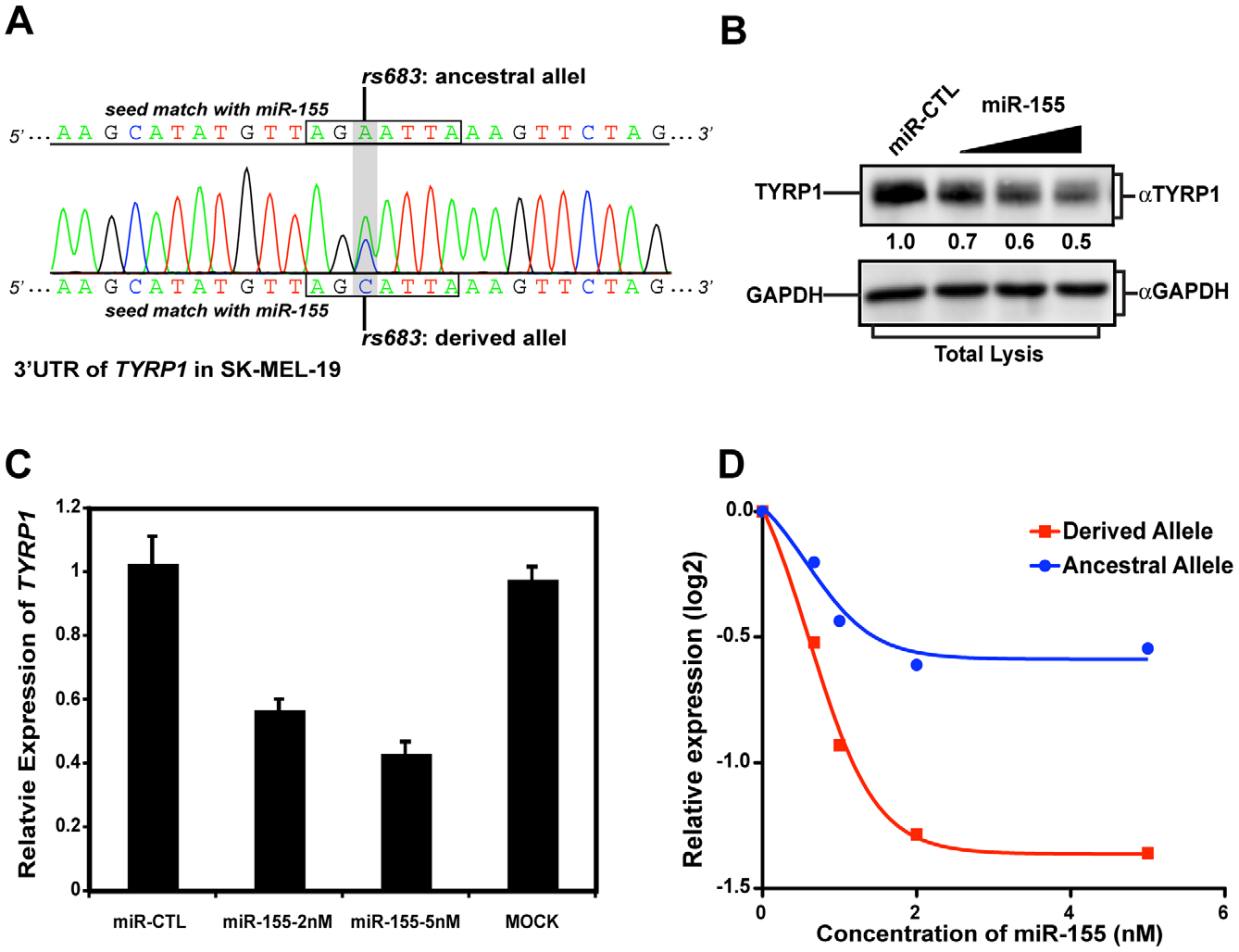
rs683 modulates endogenous *TYRP1* targeting by miR-155 in SK-MEL-19 cells. (A) Genotyping the rs683 locus in SK-MEL-19 cells. The region around rs683 was amplified from SK-MEL-19 genomic DNA and sequenced. Sequence traces (shown) revealed rs683 heterozygosity at the *TYRP1* locus, as indicated. (B) Ectopic miR-155 expression reduces *TYRP1* protein levels. *TYRP1* levels in the skin-derived melanoma cell line, SK-Mel-19 were analyzed by performing immunoblotting on cell lysates from miR-CTL transfected or cells transfected with increasing amounts of miR-155 as indicated. Densitometric quantitation of *TYRP1* levels is indicated as protein levels relative to the mock transfectants. (C) miR-155 reduces *TYRP1* mRNA levels in SK-Mel19. SK-MEL19 cells were mock transfected or transfected with the indicated miR-CTL or miR-155. mRNA was extracted and *TYRP1* levels assessed by qPCR. Results are plotted relative to miR-CTL-treated cells. Bars are the mean ± standard deviation of triplicate experiments. The differences in expression between miR-155 transfected cells and either mock or miR-CTL are statistically significant (P<0.01, Students *t* test). (D) Targeting of the derived allele by miR-155. SK-Mel-19 cells were transfected with increasing amounts of miR-155 as indicated. mRNA was then extracted and expression of the ancestral (blue) versus the derived allele (red) assessed by allele-specific TaqMan SNP qPCR. Results are plotted as the expression level of each *TYRP1* allele relative to controls (log2 transformed). Note that the transcripts carrying the derived allele were suppressed by miR-155 greater that 2-fold, whereas transcripts carrying the ancestral allele were only modestly affected.

As *TYRP1* is best known for its role in regulating human skin pigmentation, we next tested allele-specific regulation of the endogenous gene by miR-155 using a skin-derived cell line. SK-MEL-19 cells express endogenous *TYRP1*, while this gene is not expressed in many other melanoma cell lines [44]. We examined the *TYRP1* alleles in SK-MEL-19 cells and found it to be heterozygous for rs683 (Figure 3A). Next we transfected these cells with either miR-CTL or increasing amounts of miR-155, and monitored *TYRP1* protein levels by immunoblotting. This revealed that endogenous *TYRP1* protein level decreased with increasing miR-155 concentration (Figure 3B), consistent with our analysis of the heterologous luciferase reporter assay. As miRNA can suppress protein production either through destabilization, or through translational inhibition, we next performed quantitative RT-PCR (qPCR) to examine *TYRP1* mRNA levels, which were also reduced upon miR-155 transfection (Figure 3C). Finally, to demonstrate that miR-155 preferentially targets the derived allele, we employed TaqMan SNP qPCR to detect the abundance of *TYRP1* transcripts that carry either the ancestral or the derived alleles of rs683. Transfection of increasing amounts of miR-155 led to a modest reduction in the ancestral allele that contrasted the much stronger suppression observed in the derived allele (Figure 3D). Taken together, these studies establish that the derived alleles of rs683 and rs910, which are almost fixed in YRI, CHB and JPT populations (Figure 2A), introduce two additional miR-155 targets that serve to enhance miR-155-mediated suppression of *TYRP1*. In contrast, these alleles segregate at a frequency of only approximately 1/3 in CEU, with 2/3 of the population carrying the alternate ancestral allele that interferes with miR-155-mediated suppression of *TYRP1* expression.

### Population analysis of the differentiated miRNA regulation

**Figure 4:**
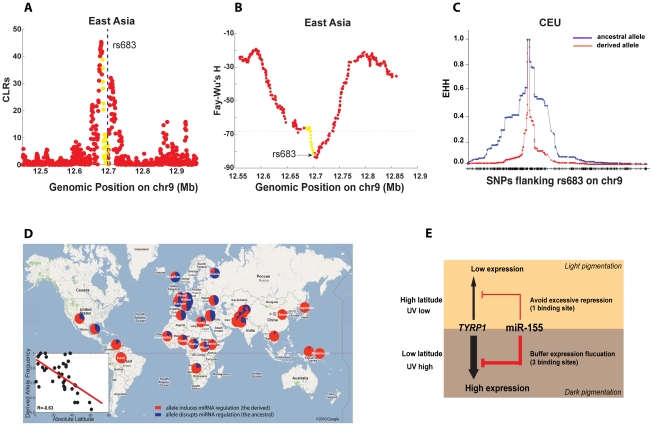
Population analysis of the miR-155 binding sites on *TYRP1*. (A–B) Statistical tests for positive selection that fixed the derived allele of rs683 in East Asians. The yellow dots represent genic position on *TYRP1*, and the flanking non-genic positions are in red. The CLR test (A, where the dotted line indicates the rs683 locus) and Fay-Wu's H test (B, where the dotted line indicates the 5% extreme value among the genome-wide SNPs) were used. (C) Recent expansion by positive selection for the ancestral allele of rs683 in CEU. The expanded haplotype homozygosity (EHH) for both the ancestral allele (in blue) and the derived allele (in red) are shown. (D) The frequencies of the derived allele of rs683 across world populations. The derived allele induces miR-155 regulation on *TYRP1*. The allele frequencies represented by the pie charts are overlaid on a customized Google Map. The inset indicates a significant negative correlation between the frequencies of the derived allele (which induces miR-155 regulation) in populations and the latitudes of population residence. (E) The model accounting for positive selection on alternate alleles of rs683 and rs910 to induce or disrupt miRNA regulation on *TYRP1* in different human populations. *TYRP1* shows higher expression in low latitudes than in high latitudes; thus selection for the derived alleles is to fix additional miRNA regulation on the highly expressed *TYRP1*, which buffers expression fluctuation. As *TYRP1* has low expression in high latitudes, then selection for the ancestral allele to disrupt miRNA regulation is a protective mechanism against excessive repression on *TYRP1*.

Previous studies suggested that *TYRP1* has been under selection in different populations [33]–[35], but none of the causal alleles were identified. We next investigated in more detail the pattern of selection that has driven miRNA site turnover in *TYRP1* between human populations. Since our analysis of the selection signature on *TYRP1* was based on inter-population comparison (*F_ST_*), we next tracked the local selection within individual populations. As shown in Figure 2A, the derived alleles of rs683 (and also the linked site rs910) are almost fixed in YRI and East Asians (CHB+JPT), and the CLR test [22] revealed a selection signature around this locus (Figure 4A for East Asians, Figure S5A for YRI), with the signal peaking around the binding sites (the dotted line). Consistent with the CLR test statistic, Fay-Wu's H statistic correspondingly showed a sharp reduction around the region of interest (Figure 4B for East Asian, Figure S5B for YRI). These signals however are absent in CEU where the derived allele is in the minor form (Figure S5C–S5D). We also did extensive analyses to explore the possibility of linkage disequilibrium (LD) between rs683 and rs910 and other known functional SNPs in the region, but could not detect any high-LD SNPs with annotated functions (see Materials and Methods and also Figure S6). Therefore it is most likely that the two miRNA binding sites mediated by rs683 and rs910 were direct targets of positive selection in YRI, CHB and JPT. Given that it is the derived states of the two SNPs that maintain miR-155 regulation (Figure 2A), positive selection, which increased the derived allele frequencies of the two *de novo* binding sites on *TYRP1* in YRI, CHB and JPT, likely reflects a requirement in these populations to induce miR-155 suppression on *TYRP1*.

In CEU the major allele is the ancestral form, and in this population there is no evidence of positive selection affecting the derived allele (Figure S5C–S5D). However, we found high extended haplotype homozygosity (EHH) [45] for the ancestral alleles of rs683 and rs910 (Figure 4C and Figure S7A), which is a sign of recent selection acting to expand the ancestral alleles in CEU. This trend is absent in other populations (Figure S7B–S7C). Further, the integrated haplotype scores (iHS) for the ancestral alleles in CEU are all above 3, substantially higher than the typical threshold of 2 used in humans for detecting loci subject to positive selection [20]. Since there are no other known functional SNPs in high LD with these SNPs in the HapMap data (see Figure S6), or in the more comprehensive set of SNPs reported in dbSNP 132 [46], which includes data from the 1000 Genomes Project [47], the observed increase in haplotype homozygosity in CEU is thus most likely explained by selection directly expanding the ancestral allele of rs683, which compromises miR-155 regulation on *TYRP1*. Taken together, these results suggest that in YRI, and particularly in CHB and JPT, the derived alleles of rs683 and rs910 have been a target of positive selection (Figure 4A and 4B, and also Figure S5A and S5B), whereas in CEU, haplotypes carrying the ancestral alleles were recently selected for, leading to an increase in haplotype homozygosity among haplotypes carrying the ancestral allele in this population (Figure 4C and Figure S7A).

Due to the significant role of *TYRP1* in modulating human pigmentation [26]–[29], a possible adaptive explanation is that the miRNA binding sites on *TYRP1* have been targeted by population-specific selection in relation to exposure to sun light (UV irradiation). We investigated this hypothesis further by extending our analysis from the 4 HapMap populations to 37 representative indigenous populations genotyped in the Human Genome Diversity Project (HGDP), spanning 650,000 common SNPs [48]. The SNP rs683 at site II is also genotyped in HGDP, which co-segregates with rs910 at site III (Figure 2A). For each population, we correlated the absolute latitude where the population resides with the derived allele frequency of rs683 (Figure 4D), and found a strong negative correlation (Pearson's *R* = −0.63). As the values for different populations might be correlated due to miration history, we cannot apply standard statistical methods to test whether the correlation is significant. However, we note that the correlation between latitude and allele frequency in rs683 is among the 1% most extreme of such correlations in the genome. This is also true if we restrict ourselves to analyses of SNPs with *F_ST_*≥0.5 (Figure S8A–S8B). Thus, the closer to the Equator that a population resides, the higher the frequency of the derived allele. There are several populations that show deviations from this trend, in particular the pygmy populations in Africa, which show less evidence of selection than the East Asian or European populations.

### Expression fine-tuning by miR-155

*TYRP1* has been suggested to be a target of positive selection, and the mode of selection on this gene is thought to be complicated [33]–[35]. Our analyses now reveal that positive selection has driven population differentiation of miR-155 regulation on this gene, providing new insights into the causes of the observed selection signatures (Figure 4). Indeed, *TYRP1* has a well-established role in regulating skin pigmentation, and its expression is ∼2.6-folder higher in Africans than in Europeans [29]. Thus the presence of selection fixing miRNA binding sites on the highly expressed *TYRP1* in Africans is a good example of *incoherent regulation*, i.e. when a miRNA represses a target gene in the direction opposing the overall outcome of all the other regulatory processes (e.g. by transcription factors) [49]–[51] (not to be confused with the *incoherent control* or *incoherent feed-forward loop* in describing generic regulatory networks). Such a network architecture is important in maintaining target protein homeostasis and in fine-tuning and buffering target protein expression. Indeed, the amount of skin pigmentation is thought to be balanced between two conflicting and UV-dependent physiological needs, the production of vitamin D and folate [52]. Hyper-pigmentation can cause vitamin D deficiency, while hypo-pigmentation can cause folate deficiency, both being tightly associated with human reproductive success. Thus, pigmentation genes are likely to be highly regulated, and gain of additional miRNA binding sites on the highly expressed *TYRP1* in Africans (in low-latitude regions) might ensure proper expression of this gene by dampening potential fluctuations that may shift its expression away from the optimal level, conferring unfavorable pigmentation phenotypes (Figure 4E). On the other hand, in high latitudes with low UV exposure, light pigmentation and low *TYRP1* expression is strongly favored. In these areas, recent selection that expanded the ancestral allele to disrupt miR-155 repression might suggest a physiological need to remove the excessive miRNA regulation on the already lowly expressed *TYRP1*, which otherwise would cause hypo-pigmentation (Figure 4E). Moreover, disrupting additional miRNA binding sites on this gene confers rapid response to external stimulus, and indeed analysis of *TYRP1* expression by solar irradiation in a variety of populations including African and European revealed that only Europeans displayed significant induction of *TYRP1* upon chronic photoexposure [29]. Therefore this mechanism might facilitate rapid adaptation to environment with elevated photoexposure. Taken together, our results reveal that the regulatory interaction between miR-155 and *TYRP1* was highly plastic during human evolution; this may serve as a physiological rheostat to optimize the expression of *TYRP1* to distinctively advantageous level in different populations, in response to differential UV radiation along the latitudes of their residence.

## Discussion

King and Wilson first proposed that most human phenotypic evolution may be due to changes in gene regulation [10]. This notion has been supported by a number of studies showing that a considerable proportion of the genetic variation underlying phenotypic human variation and human-chimpanzee differences may lie outside protein-coding regions [11]–[13]. However, genetic changes at the post-transcriptional level (e.g. regulation by miRNAs) have received little attention, and previous studies have not established a clear functional effect of alleles predicted to be under selection [7], [8]. Our study now revealed that positive selection can drive population differentiation of human miRNA regulation, suggesting that miRNA regulation could be highly evolutionarily plastic, and may contribute to human evolution.

We also found that a majority of the identified sites are in non-conserved elements revealed by genomic comparison across 17 vertebrates (quantified by phastCons score [53], which varies between 0 and 1). For example, for the validated sites in this study, their highest phastCons scores are only around 0.1, suggesting genetic novelty may arise from elements that are under relaxed selective pressure. Of note, we also scanned the known miRNA loci in our analysis, but did not find any miRNA loccus that has elevated differentiation among populations. This observation indicates that binding sites turnover may be a more prevalent mechanism in modulating miRNA regulation than changing miRNAs themselves. This notion is supported by a recent study which re-sequenced known miRNA loci among human populations and found an absolute lack of sequence diversity within the miRNA seed regions [54].

There are likely many more changes in miRNA interactions that have contributed to human adaptation than reported here. First, we only report signals relating to increased levels of population differentiation, which most likely only reveal a small fraction of the selection that has acted during human evolution since the divergence with chimpanzees. We also used a very stringent cutoff for *F_ST_* (≥0.5) in this study since we aimed to find the most extreme cases of population differentiation. Future comparative studies aimed at miRNA regulatory sites may reveal many more examples of rewired miRNA interactions. Second, our target prediction was based on TargetScanS, which uses seed matches as the principal mechanism for target recognition by miRNAs. However, other mechanisms might also exist, such as sites with central pairing [55], and it is possible that natural selection might act on sites lacking the canonical seed matches. Third, in this study, we scanned 3′UTRs of human genes for putative miRNA binding sites, however, increasing evidence has shown miRNA might also target coding regions or 5′UTRs, although repression strength is more marginal [56]. Future studies will be required to validate other putative sites identified in our study and to elucidate the underlying evolutionary significance of the selection signature.

## Materials and Methods

### SNP collections and analysis

SNP annotations were based on a previous study [17] which calculated *Fst* values for ∼3 million HapMap Phase II SNPs. When controlling for potential hitchhiking effects, we followed the protocol used in HapMap database and computed the pairwise linkage disequilibrium (LD, quantified by R2) between the SNPs in question and its flanking SNPs within 500 Kb downstream and upstream. Any SNPs having R2≥0.5 with annotated functional sites within this distance were excluded from the analysis. The function annotation of SNPs was retrieved from dbSNP 130 queried from UCSC Table Browser. Following previous protocol [20], we assigned human SNPs with ancestral alleles based on the chimpanzee reference genome (queried from UCSC Table Browser). For the 30 SNPs of particular interest, we also confirmed its ancestral allele by comparing with the reference genomes of orangutan and rhesus macaque (Figure S1B).

Allele frequencies of rs683 in world populations were collected from the Human Genome Diversity Project [48], and were extracted from the UCSC Table Browser. For each population genotyped, we extracted its absolute latitude (the absolute value of the latitude), which indicates the angle of a location from the Equator rather than relative north and south. Among the genotyped populations, we used Han to represent ∼92% of Chinese population, and excluded the Chinese minorities from our analysis due to their complicated ethnohistorical characteristics and migration histories. We also excluded Yakut as they are very recent migrants approximately 1,000 years ago, with an effective female population size of only 150 individuals [57]. This is because population migration might distort our analysis of long-term selection in particular population residence.

The iHS values and Fay and Wu's H for HapMap populations were extracted from Haplotter (http://haplotter.uchicago.edu/). CLR test was performed using SweepFinder [22] by setting the background site frequency spectrum estimated from all SNPs across the genome. SNPs from HapMap (rel.27) were subject to this analysis.

### Analysis of linkage disequilibrium

To explore the possibility that rs683 and rs910 changed frequency due to hitchhiking with some other functional variants on this gene, we analyzed all the known HapMap SNPs on *TYRP1* in YRI and CEU, and computed their linkage disequilibrium (LD) R2 with rs683 using PGEToolbox [58] (Figuere S8). We also considered SNPs in the 5-Kb upstream region of TYRP1. Most SNPs that showed significant LD with rs683 are intronic and do not overlap with any annotated splice sites, while two loci in the 5′ upstream region showed only weak LD. Although one missense SNP was found, it was not linked with rs683 and displayed an extremely low LD. In contrast, two SNPs rs2762464 and rs1063380, located in the 3′UTR of TYRP1 (Figure S6), were within a strong linkage disequilibrium region of rs683 and rs910, consistent with their close physical proximity (<300 bp away). However, neither has any known mechanistic association with *TYRP1* and none of the SNPs mediates interaction with known miRNAs. Similarly in CHB and JPT, we did not find any known functional variants on *TYRP1* in strong LD with rs683. We also expanded the analysis from HapMap SNPs to dbSNP132 by querying the UCSC Table Browser, which includes SNPs genotyped in the 1000 Genomes Project [47], and did not find any annotated functional variants in high LD with rs683.

### Compilation of miRNAs and the predicted targets

We first extracted all SNPs annotated to be in 3′ UTR of human genes, which were annotated by HapMap (rel.27 for all populations), and all the SNPs are polarized to the plus strand. These 3′UTR SNPs were then mapped onto the 3′UTR sequences of RefSeq transcripts (downloaded from UCSC Table Browser as of July, 2010), and we retained the longest transcripts when multiple sequences are annotated under the same transcript. Sequences with inconsistent annotations were discarded from our analysis. With this mapping procedure, we then generated a set of polymorphic 3′UTR segments, which is a 15 nucleotide window centered at the SNP position. Therefore for each SNP, sequences within the window will present twice each carrying the alternate alleles. TargetScanS [15] was then implemented to scan the collection of the polymorphic 3′UTR segments, and the predicted sites were then identified, which encompass a SNP, one of whose alleles does not interact with any miRNA while the other is miRNA-interacting. Prediction confidence was determined by context scores assigned the prediction program and we considered confident sites if their context scores no more than −0.2 (Figure S1A). We considered 545 miRNA families deposited in TargetScanS (Table S4). For experimental validation, we further scanned the putative miRNA sites in fine solution by allowing a 6-mer match.

### DNA constructs and reagents

All the constructs for this study were derived from the pMIR-REPORTER (Ambion). Human 3′UTR sequences in this study were amplified from genomic DNA of HEK293T cells using PCR. The PCR products were subcloned into pMIR-REPORTER vector. Overlapping PCR and QUICKCHANGE II XL Site-Directed Mutagenesis Kits (Agilent) were used to generate the mutants of 3′UTRs containing different SNPs and deletions of miRNA target sites. pMIR-REPORTER β-galactosidase vector was used as the transfection control. G3R is a gift from Dr. Burton Yang' lab at the University of Toronto, which has the coding sequence of the chicken versican G3 domain in the pMIR-REPORTER vector, and was used as a negative control. All the synthesized miRNA mimics and the mimic negative control were purchased from Dharmacon. miR-CTL in this study is a negative control with sequence from a C.elegans miRNA cel-miR-67, which has minimal sequence identity with miRNAs in human, mouse and rat (by Dharmacon).

### Cell transfection, luciferase assay, immunoblotting, and qPCR

HEK293T cells were grown in high glucose DMEM containing 10% FBS (Thermo). They were transiently transfected with DNA constructs and miRNA mimics at 40% confluency in 24-well plates using the calcium-phosphate precipitation method. Cells were lysed 48 hours after transfection, and the activities of firefly luciferase and β-galactosidase of total cell lysates were determined using the Firefly Luciferase Assay System (Promega) and the β-gal assay previously described, respectively. To obtain the relative activity, the activity of the firefly luciferase was first normalized to the activity of β-galactosidase to obtain normalized firefly luciferase activity (nFFLuc), and the data were then determined using the following formula: Relative Activity = log2(nFFLucmiR-155/nFFLucmiR-CTL), where nFFLucmiR-155 or nFFLucmiR-CTL is the nFFLuc in the presence of mir-155 mimics or mimic negative control. The concentration of miR-155 was increased as indicated in the figures (Figure 1C and 1D, Figure 2C–2E, and Figure 3B–3D), with the concentration of miR-CTL being 2 nM. If Relative Activity was positive, we manually set it to be 0. All the positive Relative Activity are no more than 0.13.

SK-MEL-19 cells were grown in RPMI 1640 (GIBCO) containing 10% FBS and antibiotics. For genotyping, the lysates of SK-MEL-19 cells were subjected to PCR. The forward primer starts from 116 bps upstream of rs683 whereas the reverse primer starts from 118 bps downstream of rs683. The PCR products were cleaned and sent to DNA sequencing using the forward primer. The sequencing spectra were processed and analyzed using MacVector. SK-MEL-19 cells were transiently transfected with miRNA mimics following the instructions of the RNAi MAX transfection kit (Invitrogen). For immunoblotting analysis, cells lysates were collected 40 hours after transfection. The antibodies used for immunoblotting analysis are: TYRP1 (sc-10443, Santa Cruz) and GAPDH (G9545, Sigma). The ΔCT values were obtained by comparing the amplification of the target cDNA to that of HPRT. The TapMan SNP qPCR kit for rs683 (C_3119206_10, AB Biosystems) was used to analyze the expression of the TYRP1 transcripts carrying its alternate alleles. This kit included two probes, one conjugated with VIC fluorescence dye to monitor transcripts carrying the ancestral allele and the other conjugated with FAM fluorescence dye to detect the derived allele. To evaluate the cross-hybridization between the probes, a pilot qPCR analysis was performed for the constructs A and B in Fig. 2B, each carrying the ancestral and derived alleles, respectively (Figure S9). A total of 8 samples by mixing the construct A and B were prepared as the following ratios of A to B: 1∶0, 1∶1, 1∶2, 1∶4, 4∶1, 2∶1, 0∶1. The total DNA concentrations of the 8 samples were constant with 0.1 ug/ul. The same amount of DNA were then taken from these 8 samples individually to dilute 1000 times in water and these 8 diluted samples were subject to TaqMan SNP qPCR analysis according to the instructions of the kit. Expected Relative Fraction refers to the fraction of the construct in the mixed samples relative to the construct in the sample without mixing the other construct. Observed Relative Fraction was obtained using qPCR ΔCt of the construct in the mixed sample relative to the ΔCt of the construct in the sample without mixing the other construct. Using Observed and Expected Relative Fractions, two regression lines were plotted for the FAM and VIC signals to determine the effects of cross-hybridization between the probes. We then used these probes to detect the relative expression of TYRP1 transcripts carrying different alleles in the SK-MEL-19 cells, and probe intensities after miR-155 transfection were obtained after normalizing to the data points by transfecting miR-CTL.

## Supporting Information

Figure S1 Identifying the predicted miRNA binding sites showing population differentiation. (A) Distribution of the context score among the predicted sites affected by SNPs. Prediction confidence was quantified by context score where more negative score indicates more confident prediction. Based on the distribution, a site considered confident if its context score no more than −0.2. (B) Hierarchical clustering of the ancestral allele frequencies in the 4 populations, YRI, CEU, CHB and JPT. The SNPs were clustered into 4 blocks as indicated from block I to block IV, with highly differentiated patterns among the 4 populations. The two SNPs (rs683 and rs910) on *TYRP1* are indicated in block III.
(TIF)

Figure S2 (A–D) The CLR test localizes selection signal on the polymorphic miRNA binding sites (indicated in blue below the X-axis in each panel). The dotted lines indicate the loci for each SNP analyzed.
(TIF)

Figure S3 (A–D) The luciferase reporter assay in HEK293T revealed four genes did not respond to their predicted miRNA regulators. The ancestral alleles are shaded in blue whereas the derived alleles are shaded in red. The reporter constructs were individually transfected into HEK293T cells with miR-CTL or with the predicted miRNAs as indicated. The luciferase relative activities were obtained and analyzed as described in Figure 1.
(TIF)

Figure S4 Lack of selection signal on the derived allele of rs7198 in YRI (A–B) and CEU (C–D) revealed by CLR test (A–C, where the dotted lines indicate rs7198 locus) and Fay-Wu's H test (B–D, where the dotted line indicates the 5% extreme value among the genome-wide SNPs). The derived allele in CEU is 0.67, whose H is marginally significant as shown in (D), but is not supported by CLR test (C). The threshold of statistical significance was not shown in (B) as all the values were insignificant, far beyond the threshold.
(TIF)

Figure S5 Statistical tests for positive selection on the derived allele of rs683 in YRI (A–B) and CEU (C–D). CLR and Fay-Wu's H tests consistently localized the selection signal around the rs683 locus in YRI (A–B), but not in CEU (C–D). The dotted line for H statistic indicates the 5% extreme value among the genome-wide SNPs.
(TIF)

Figure S6 Linkage disequilibrium between rs683 and all other known variants on *TYRP1* and its 5 kb upstream in YRI (the first row) and CEU (the second row). Each column represents one SNP, and different genomic regions are separated by red vertical bars, including a 5 Kb upstream region. The brighter color indicates higher association (*R^2^*) of a given SNP with rs683. The 6^th^ SNP rs12001162 is absent in HapMap CEU population. In CEU and YRI, the strongly linked loci with rs683 are intronic and do not overlap with known splice sites. The only missense SNP is not associated with rs683. The two other SNPs in close proximity to rs683 and rs910 on the 3′ UTR do not interact with any known human miRNAs.
(TIF)

Figure S7 Plots of the extended haplotype homozygosity (EHH) in the 0.5 Mb region centering at rs683 in CEU (A), East Asia (B) and YRI (C). Haplotypes carrying the ancestral or the derived allele of rs683 are in blue or red, respectively.
(TIF)

Figure S8 Histograms of the Pearson's correlation coefficients between the absoulte latitides of HGDP populations and the derived frequencies of SNPs across the genome (A) or SNPs with high-*F_ST_* (≥0.5) (B). In either case the correlation derived by rs683 is among the extreme 1% of all the SNPs analyzed (the red bar).
(TIF)

Figure S9 Evaluation of the cross-hybridization between probes for the TaqMan SNP qPCR assay for rs683. In this assay one probe conjugated with VIC fluorescence dye monitors the ancestral allele (the blue circles) and the other probe conjugated with FAM fluorescence dye detects the derived allele (the red squares). Constructs A and B used in Fig. 2B were used for this evaluation, carrying the ancestral and derived alleles, respectively. 8 samples with the mixed construct A and B were prepared as the following ratio of A to B: 1∶0, 1∶1, 1∶2, 1∶4, 4∶1, 2∶1, 0∶1, with the constant total DNA concentrations for the 8 samples at 0.1 ug/ul. Expected Relative Fraction refers the fraction of the construct in the mixed samples relative to the construct in the sample without mixing the other construct. Observed Relative Fraction was obtained using qPCR ΔCt of the construct in the mixed sample relative to the ΔCt of the construct in the sample without mixing the other construct. Using data points for Observed (x-axis) and Expected (y-axis) Relative Fractions, two regression lines were plotted for the FAM (the red line for the derived allele) and VIC (the blue line for the ancestral allele) signals respectively. The slopes of the blue and red lines are 0.91 and 0.94, respectively, very close to 1. R square values for both regression lines are 0.95 and 0.92 respectively, indicating a lack of cross-hybridization between probes for the alternate alleles of rs683.
(TIF)

Table S1 The 30 SNPs and 26 human genes identified in this study. The confidence of miRNA target prediction and the associated selection statistics are also shown. Alternative transcripts of these genes as annotated by RefSeq are shown individually. Note that more than one miRNAs can share one target site and thus a single SNP can impact the regulation by multiple microRNAs, for example the SNP rs9893667 on gene NM_006380 (APPBP2) can influence the target site of hsa-miR-362-3p, hsa-miR-329, and hsa-miR-603 (highlighted in bold).
(XLS)

Table S2 Summary of molecular functions of the identified genes. Annotations were queried from DAVID (http://david.abcc.ncifcrf.gov). In this table, each gene is annotated with its OMIM disease association, gene ontology and KEGG pathways. Note C4orf46 is a hypothetical gene with no function annotation.
(XLS)

Table S3 Functional annotation of the 7 genes selected for experimental validation.
(XLS)

Table S4 miRNA families considered in this study. miRNAs were grouped into families based on their seed identity. The miRNA family information was deposited in TargetScanS.
(XLS)
