# Supplementary figures and images for: Evidence for Positive Selection on a Number of MicroRNA Regulatory Interactions during Recent Human Evolution
Source: PLoS Genet. 2012 Mar 22;8(3):e1002578. doi: 10.1371/journal.pgen.1002578 (PMC3310733; doi:10.1371/journal.pgen.1002578)

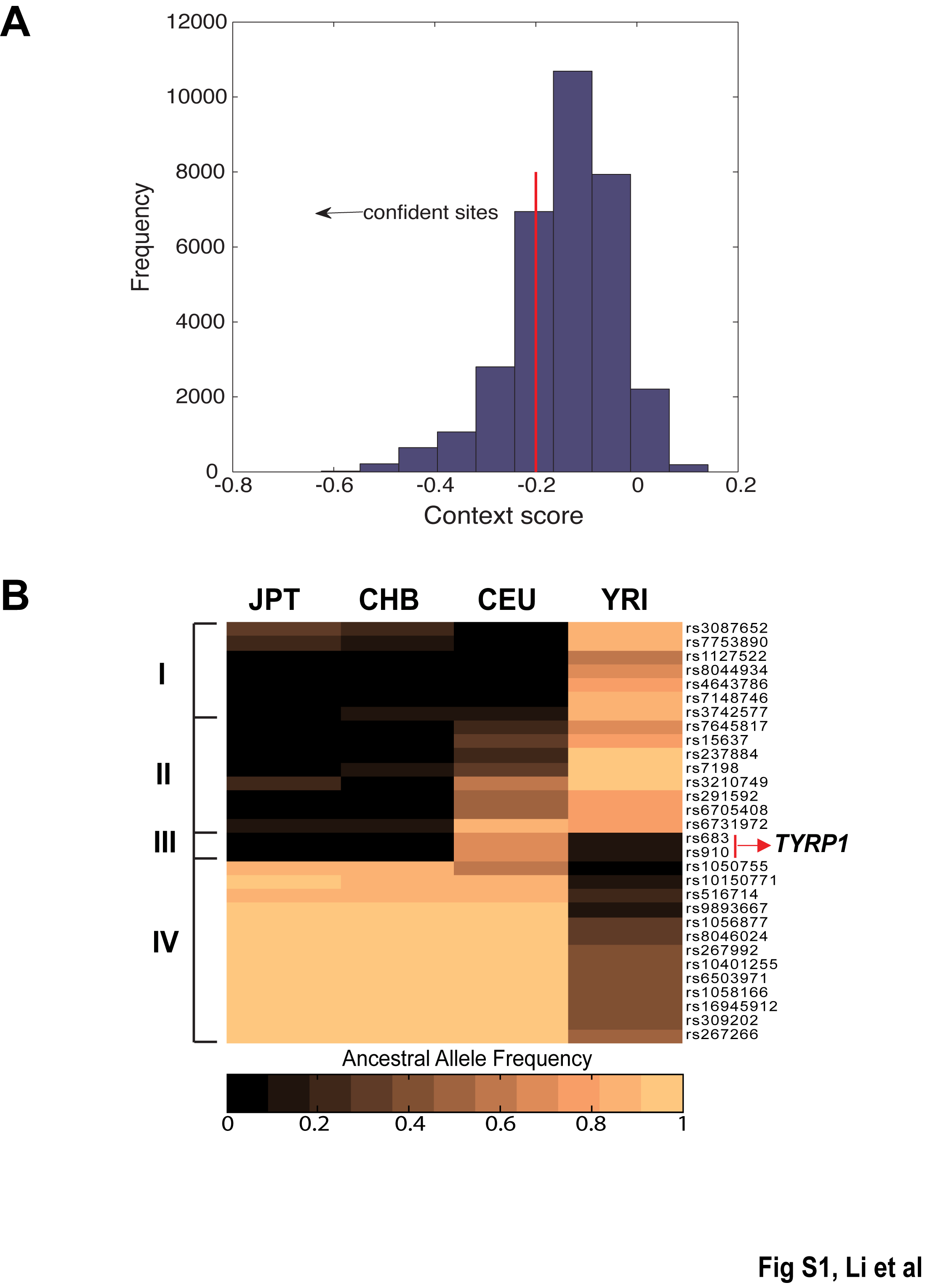

Supplement: Figure S1 — Identifying the predicted miRNA binding sites showing population differentiation. (A) Distribution of the context score among the predicted sites affected by SNPs. Prediction confidence was quantified by context score where more negative score indicates more confident prediction. Based on the distribution, a site considered confident if its context score no more than −0.2. (B) Hierarchical clustering of the ancestral allele frequencies in the 4 populations, YRI, CEU, CHB and JPT. The SNPs were clustered into 4 blocks as indicated from block I to block IV, with highly differentiated patterns among the 4 populations. The two SNPs (rs683 and rs910) on TYRP1 are indicated in block III. (TIF) [file pgen.1002578.s001.tif]

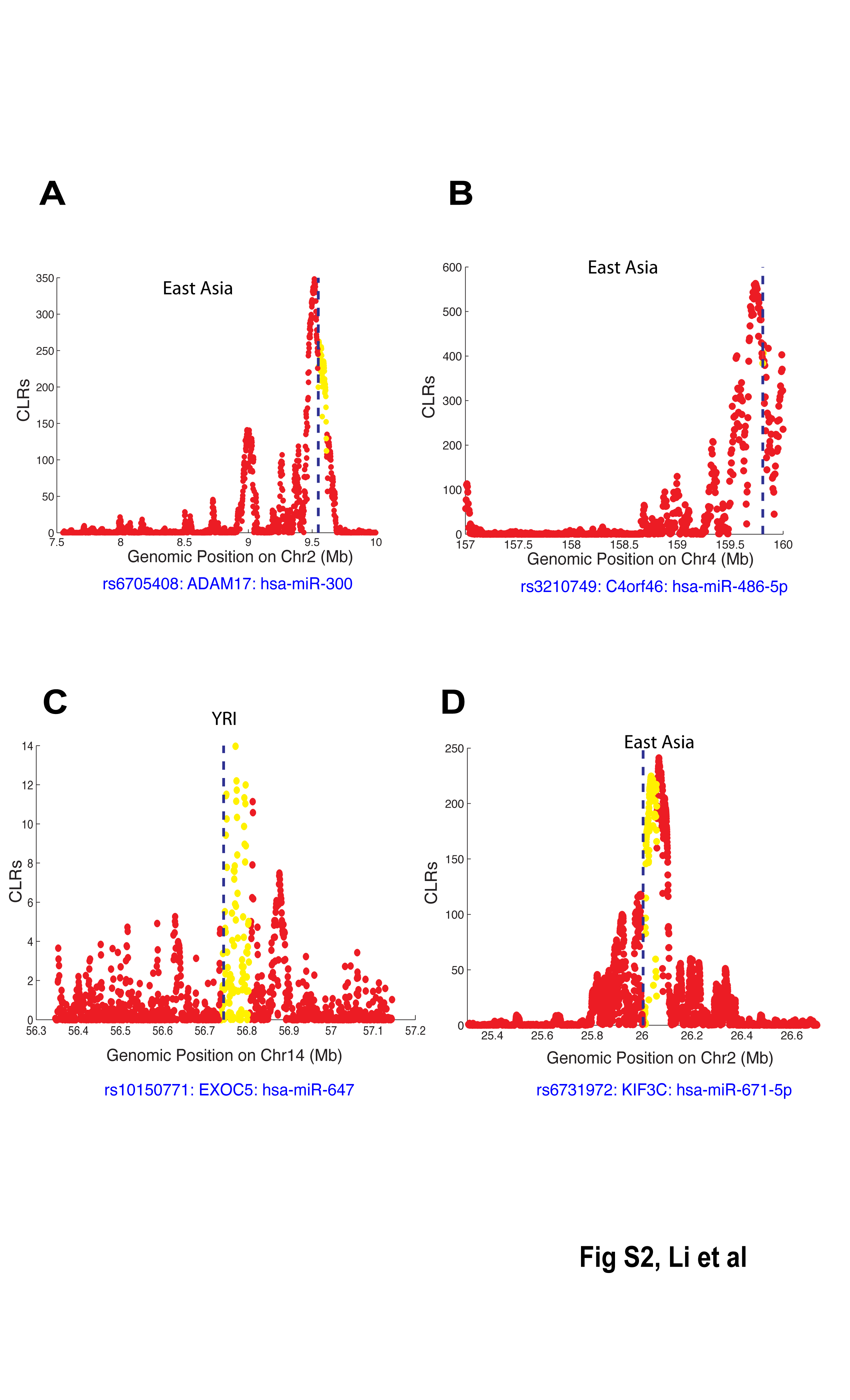

Supplement: Figure S2 — (A–D) The CLR test localizes selection signal on the polymorphic miRNA binding sites (indicated in blue below the X-axis in each panel). The dotted lines indicate the loci for each SNP analyzed. (TIF) [file pgen.1002578.s002.tif]

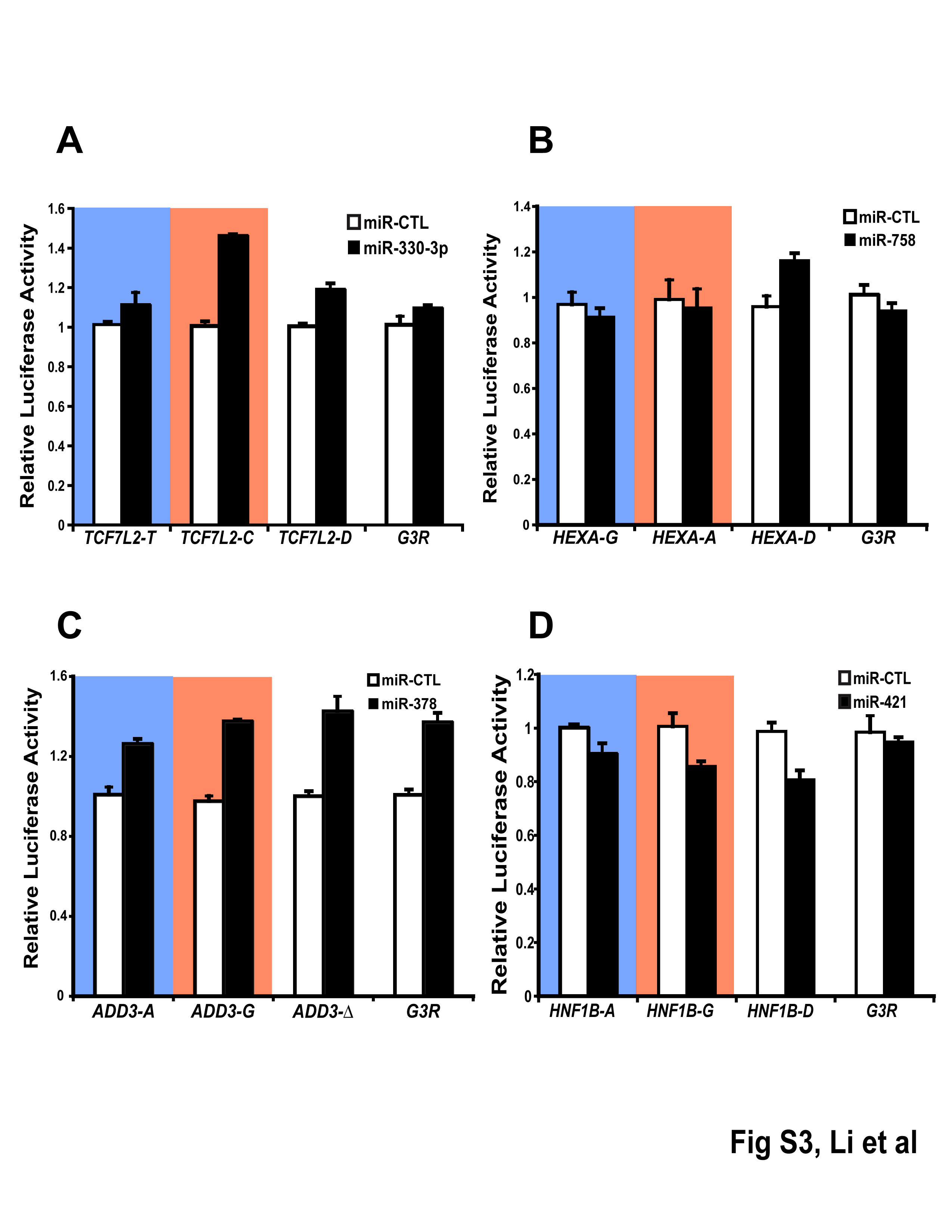

Supplement: Figure S3 — (A–D) The luciferase reporter assay in HEK293T revealed four genes did not respond to their predicted miRNA regulators. The ancestral alleles are shaded in blue whereas the derived alleles are shaded in red. The reporter constructs were individually transfected into HEK293T cells with miR-CTL or with the predicted miRNAs as indicated. The luciferase relative activities were obtained and analyzed as described in Figure 1. (TIF) [file pgen.1002578.s003.tif]

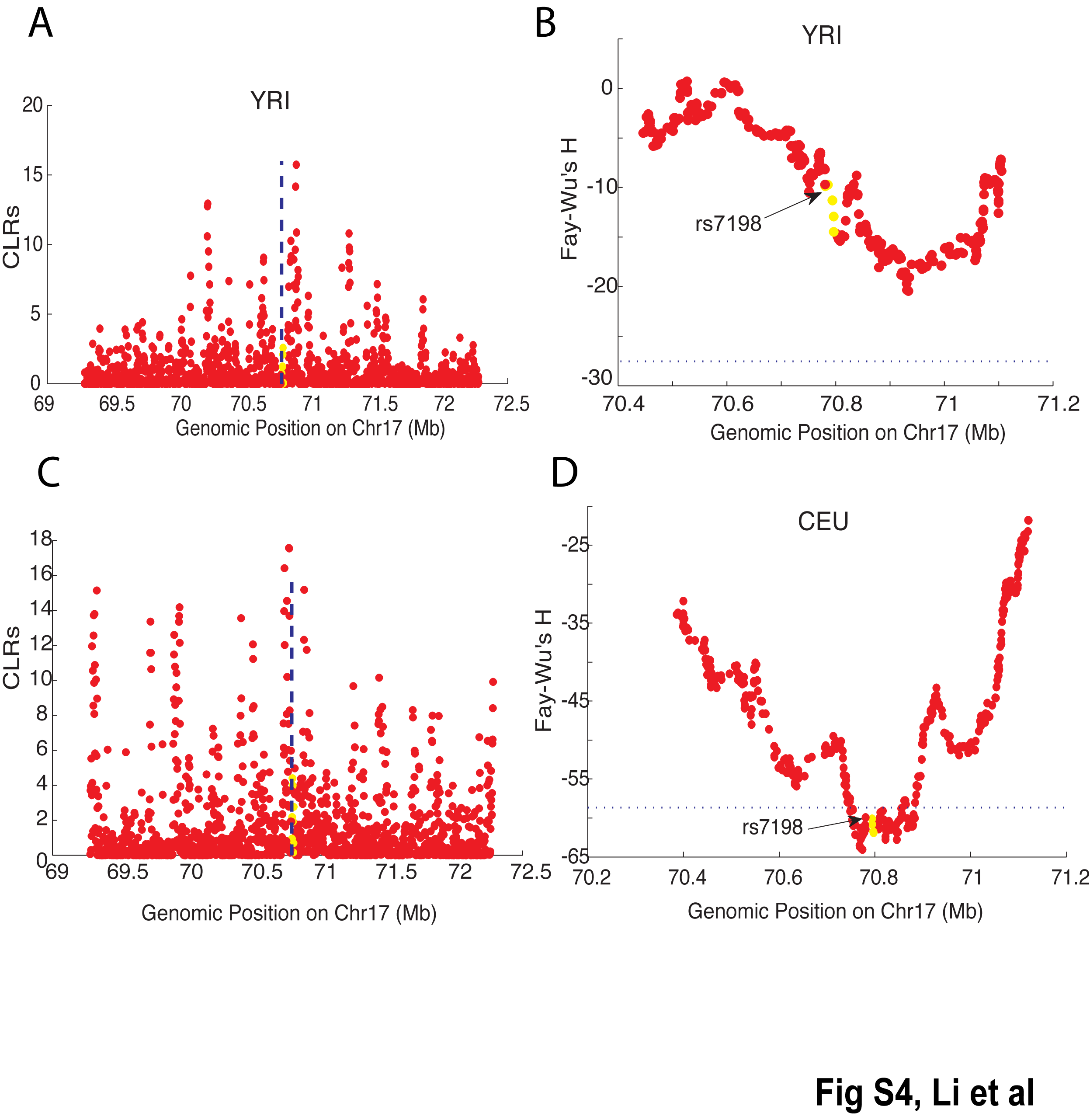

Supplement: Figure S4 — Lack of selection signal on the derived allele of rs7198 in YRI (A–B) and CEU (C–D) revealed by CLR test (A–C, where the dotted lines indicate rs7198 locus) and Fay-Wu's H test (B–D, where the dotted line indicates the 5% extreme value among the genome-wide SNPs). The derived allele in CEU is 0.67, whose H is marginally significant as shown in (D), but is not supported by CLR test (C). The threshold of statistical significance was not shown in (B) as all the values were insignificant, far beyond the threshold. (TIF) [file pgen.1002578.s004.tif]

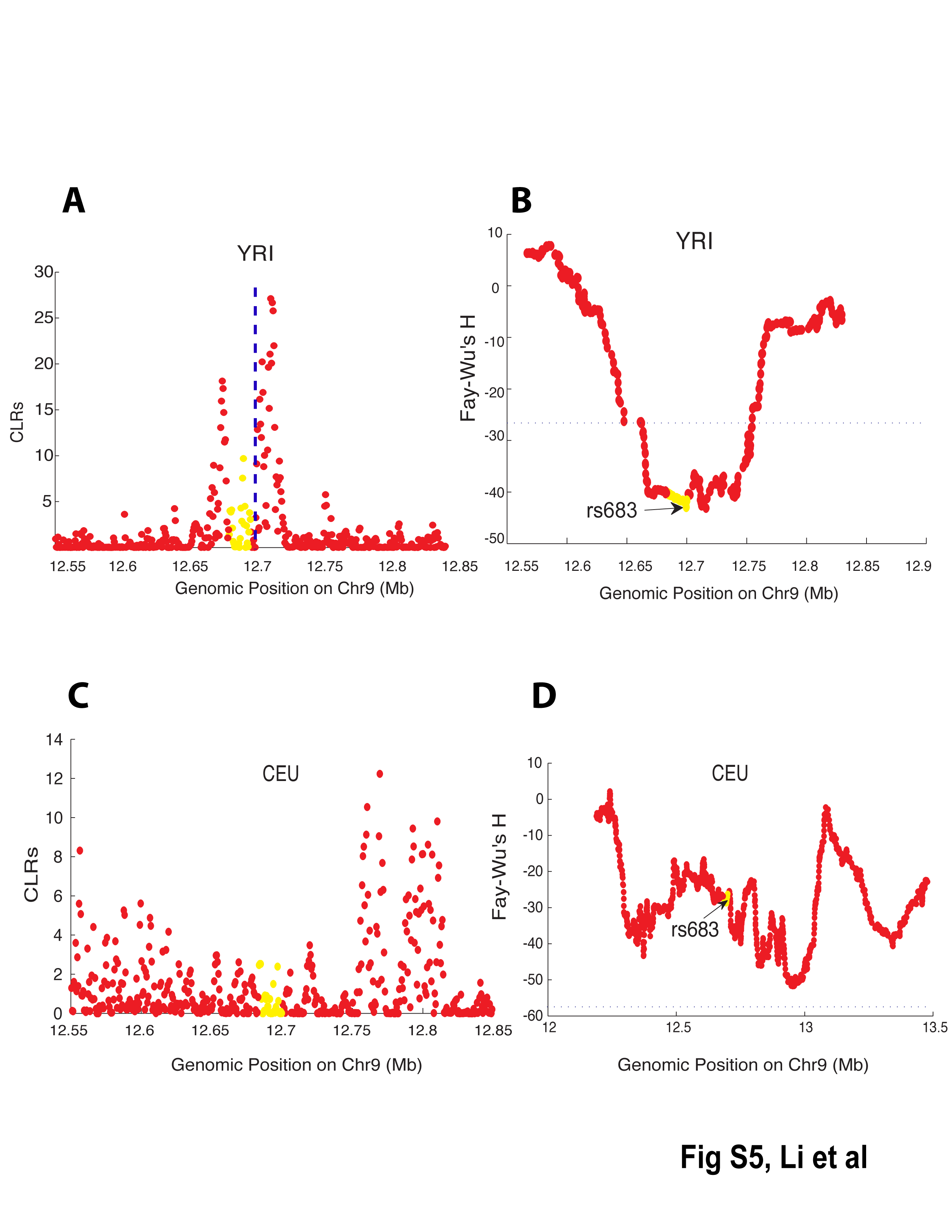

Supplement: Figure S5 — Statistical tests for positive selection on the derived allele of rs683 in YRI (A–B) and CEU (C–D). CLR and Fay-Wu's H tests consistently localized the selection signal around the rs683 locus in YRI (A–B), but not in CEU (C–D). The dotted line for H statistic indicates the 5% extreme value among the genome-wide SNPs. (TIF) [file pgen.1002578.s005.tif]

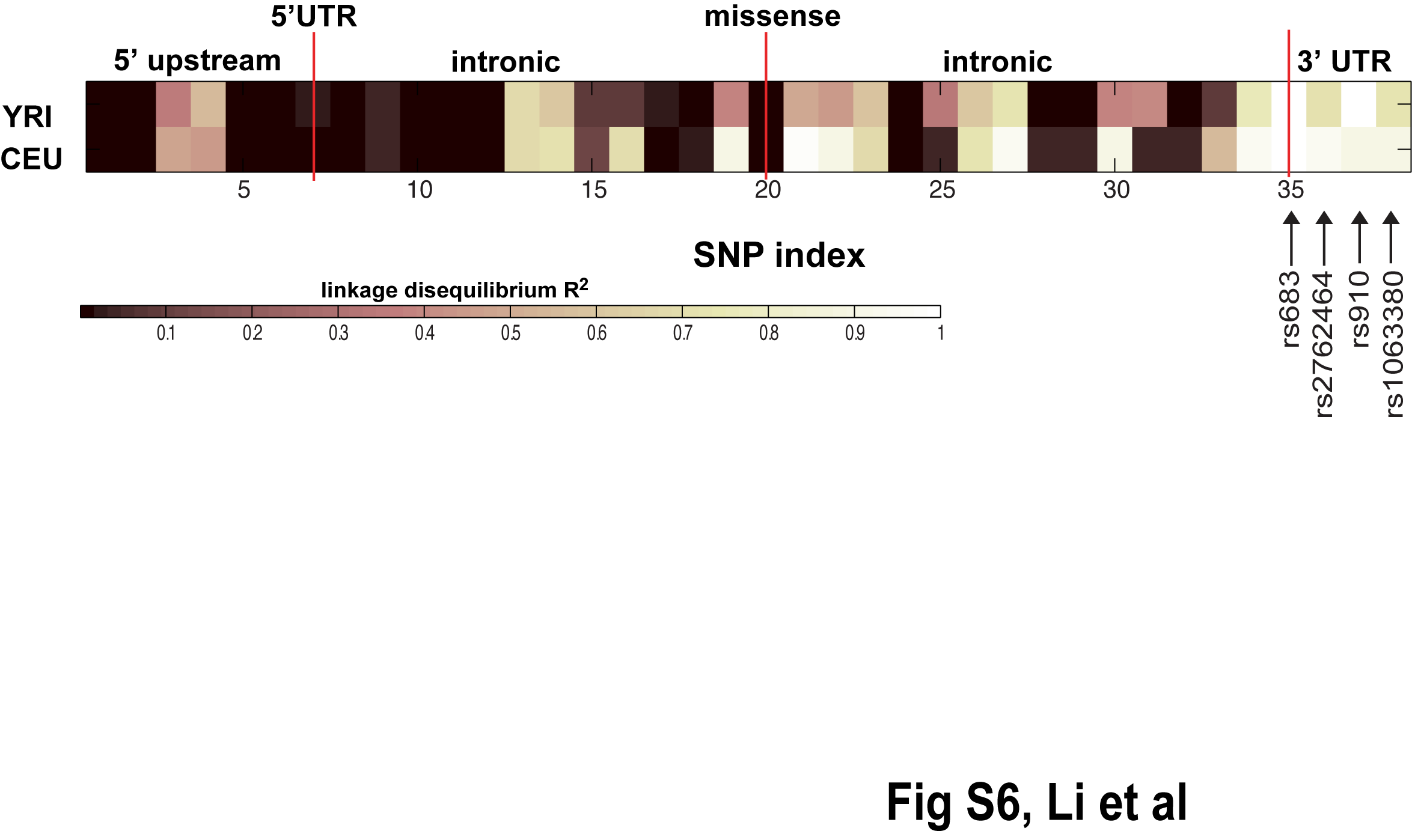

Supplement: Figure S6 — Linkage disequilibrium between rs683 and all other known variants on TYRP1 and its 5 kb upstream in YRI (the first row) and CEU (the second row). Each column represents one SNP, and different genomic regions are separated by red vertical bars, including a 5 Kb upstream region. The brighter color indicates higher association (R2) of a given SNP with rs683. The 6th SNP rs12001162 is absent in HapMap CEU population. In CEU and YRI, the strongly linked loci with rs683 are intronic and do not overlap with known splice sites. The only missense SNP is not associated with rs683. The two other SNPs in close proximity to rs683 and rs910 on the 3′ UTR do not interact with any known human miRNAs. (TIF) [file pgen.1002578.s006.tif]

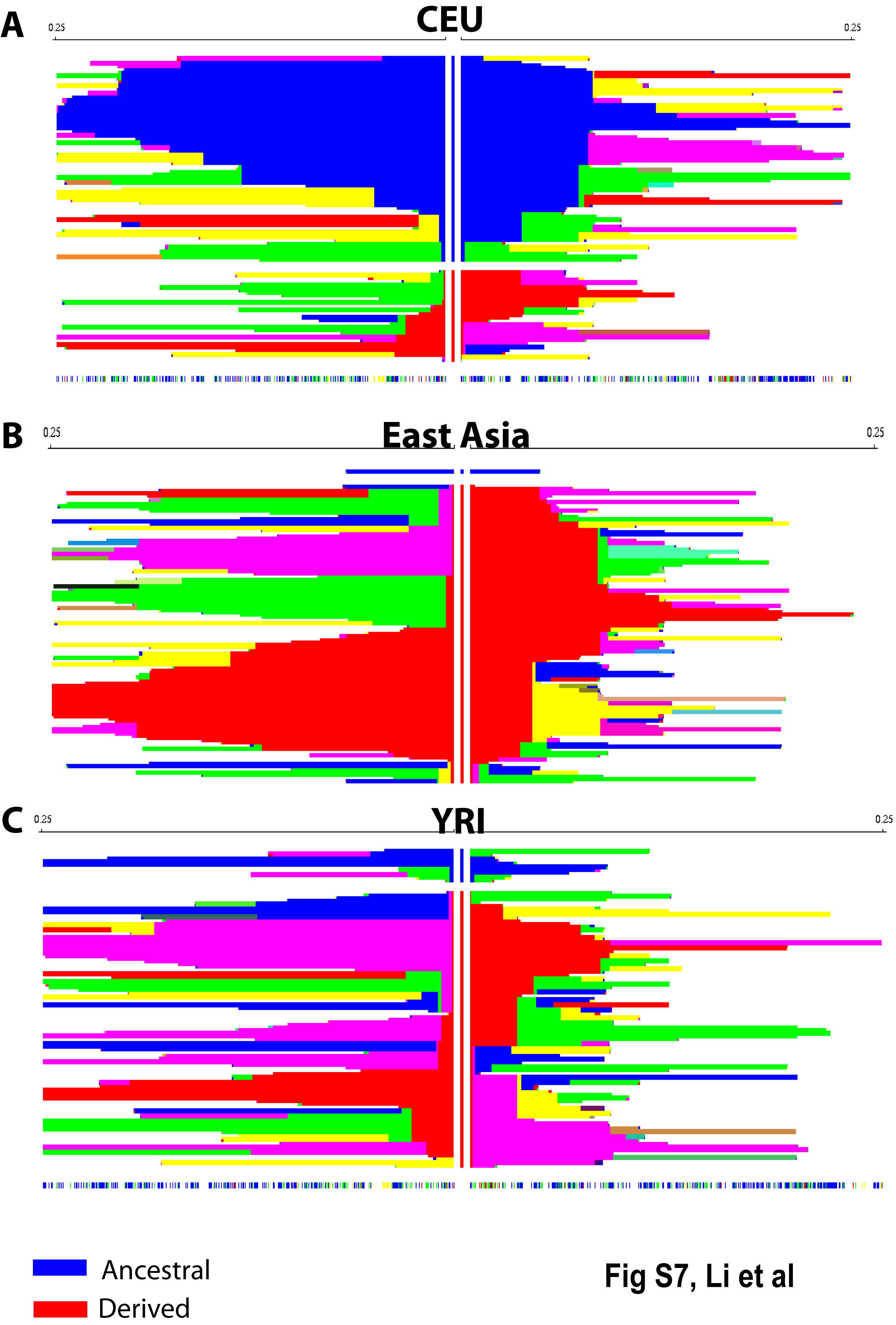

Supplement: Figure S7 — Plots of the extended haplotype homozygosity (EHH) in the 0.5 Mb region centering at rs683 in CEU (A), East Asia (B) and YRI (C). Haplotypes carrying the ancestral or the derived allele of rs683 are in blue or red, respectively. (TIF) [file pgen.1002578.s007.tif]

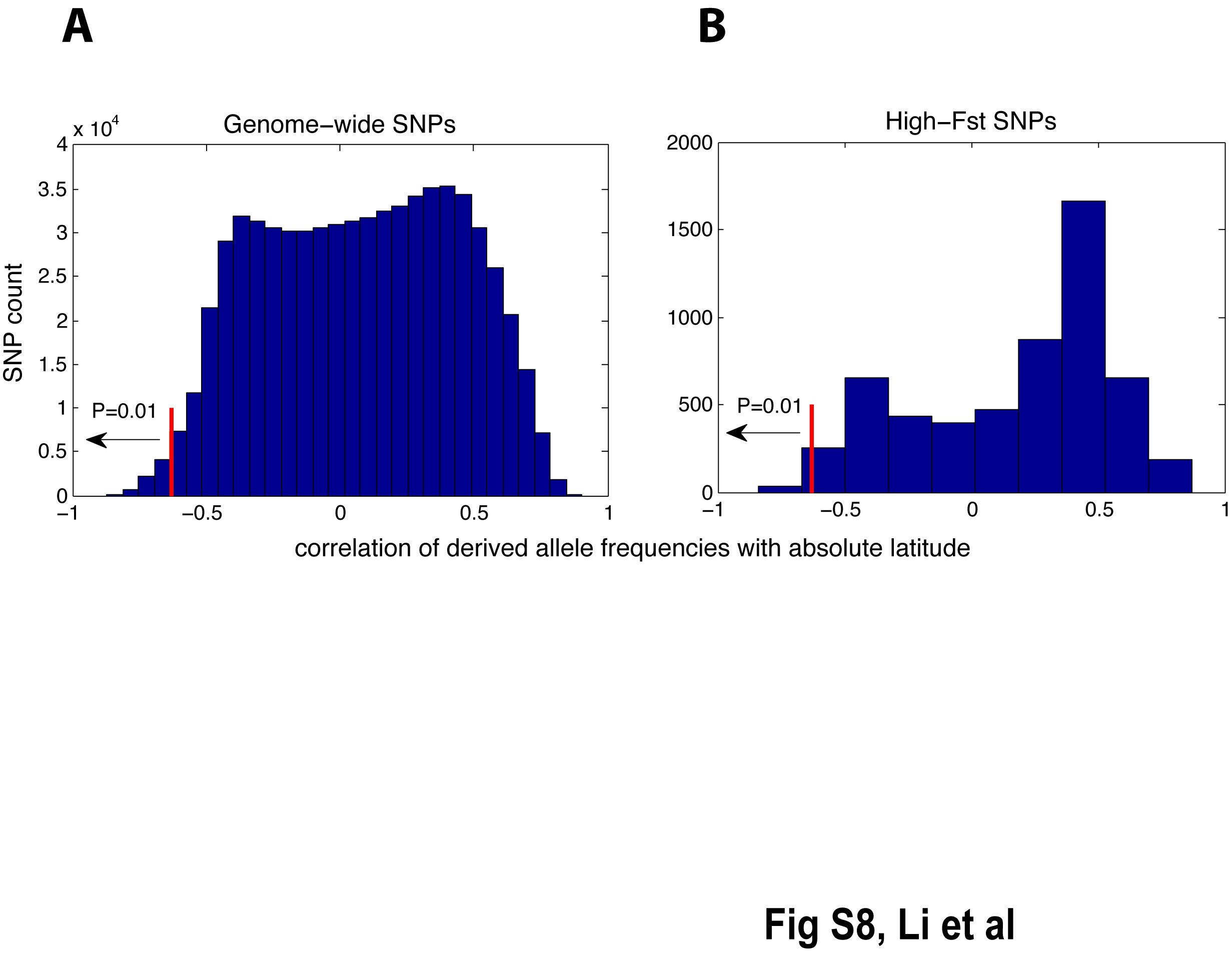

Supplement: Figure S8 — Histograms of the Pearson's correlation coefficients between the absoulte latitides of HGDP populations and the derived frequencies of SNPs across the genome (A) or SNPs with high-FST (≥0.5) (B). In either case the correlation derived by rs683 is among the extreme 1% of all the SNPs analyzed (the red bar). (TIF) [file pgen.1002578.s008.tif]

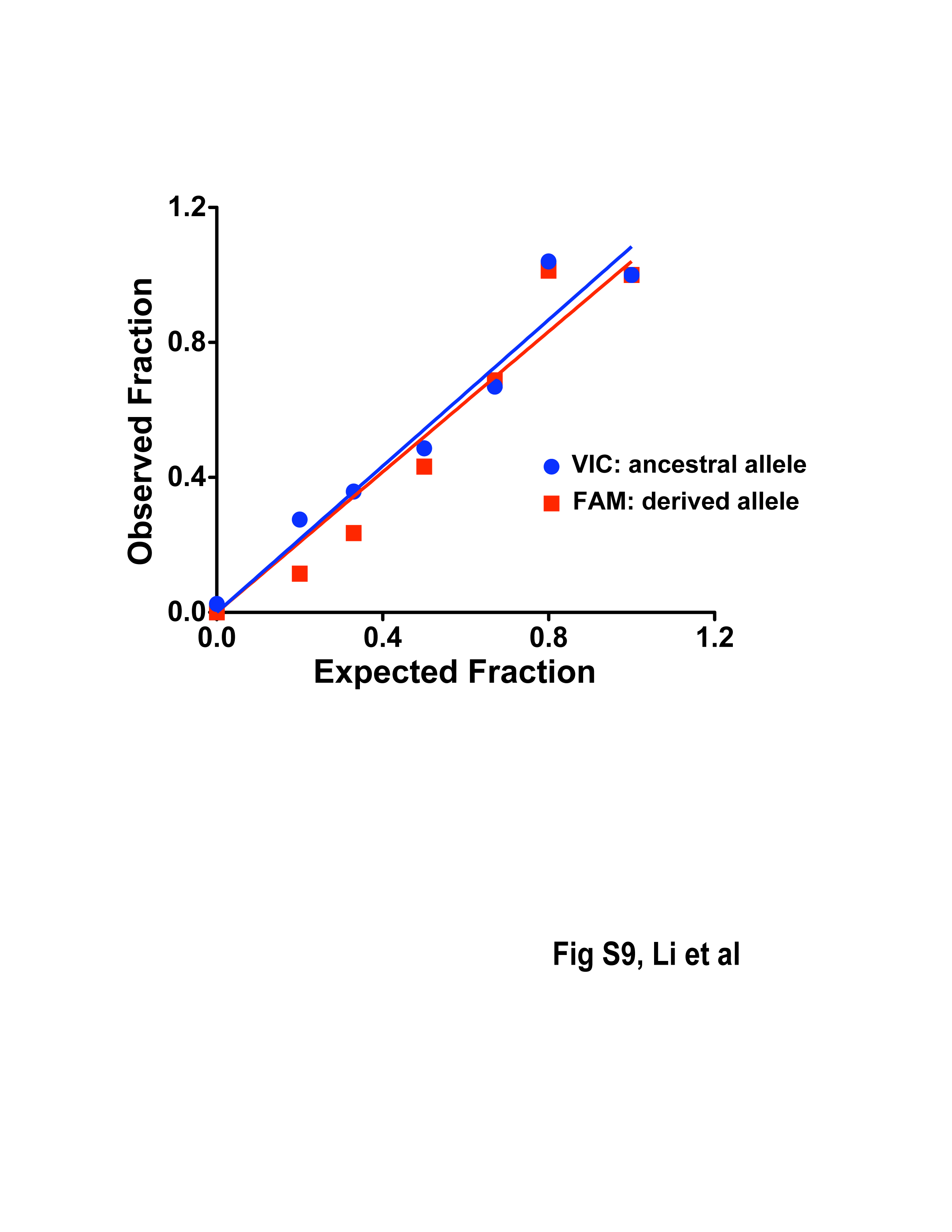

Supplement: Figure S9 — Evaluation of the cross-hybridization between probes for the TaqMan SNP qPCR assay for rs683. In this assay one probe conjugated with VIC fluorescence dye monitors the ancestral allele (the blue circles) and the other probe conjugated with FAM fluorescence dye detects the derived allele (the red squares). Constructs A and B used in Fig. 2B were used for this evaluation, carrying the ancestral and derived alleles, respectively. 8 samples with the mixed construct A and B were prepared as the following ratio of A to B: 1∶0, 1∶1, 1∶2, 1∶4, 4∶1, 2∶1, 0∶1, with the constant total DNA concentrations for the 8 samples at 0.1 ug/ul. Expected Relative Fraction refers the fraction of the construct in the mixed samples relative to the construct in the sample without mixing the other construct. Observed Relative Fraction was obtained using qPCR ΔCt of the construct in the mixed sample relative to the ΔCt of the construct in the sample without mixing the other construct. Using data points for Observed (x-axis) and Expected (y-axis) Relative Fractions, two regression lines were plotted for the FAM (the red line for the derived allele) and VIC (the blue line for the ancestral allele) signals respectively. The slopes of the blue and red lines are 0.91 and 0.94, respectively, very close to 1. R square values for both regression lines are 0.95 and 0.92 respectively, indicating a lack of cross-hybridization between probes for the alternate alleles of rs683. (TIF) [file pgen.1002578.s009.tif]
